# The role of resistance training and creatine supplementation on oxidative stress, antioxidant defense, muscle strength, and quality of life in older adults

**DOI:** 10.3389/fpubh.2023.1062832

**Published:** 2023-05-02

**Authors:** Ehsan Amiri, Dariush Sheikholeslami-Vatani

**Affiliations:** Department of Sport Sciences, University of Kurdistan, Sanandaj, Iran

**Keywords:** creatine, antioxidant defense, aging, quality of life, muscular endurance

## Abstract

**Background:**

The aim of this study was to evaluate the effect of resistance training (RT) with creatine monohydrate supplementation (CS) on serum levels of *8-hydroxydeoxyguanosine* (8-OHdG), malondialdehyde (MDA), glutathione peroxidase (GPX), and total antioxidant capacity (TAC) in older adults.

**Objectives:**

This study evaluated the effect of resistance training with creatine monohydrate supplementation on oxidative stress and antioxidant defense, muscle strength and quality of life in older adults.

**Methods:**

We examined 45 non-athlete volunteer older men and women (mean, 68.1 ± 7.2  years old), were randomly selected and divided into three groups of 15: RT with creatine supplementation (RT + CS), RT with placebo (RT + P) and control group. RT protocol was performed for 10  weeks, three sessions per week. Creatine supplement was taken daily at a dose of 0.1 g/kg of body weight, while the placebo group consumed the same amount of starch. Fasting blood samples were taken before the start of program and at the end of the RT period.

**Results:**

In the training groups, after 10  weeks of RT, a significant decrease in MDA and 8 - OHDG as well as a significant increase in serum levels of GPX and TAC were observed (in all cases, *p* =  0.001). In addition, creatinine levels were enhanced in the RT + CS (*p* =  0.014). Training intervention also improved quality of life and muscle strength in the experimental groups (*p* =  0.001), although muscle strength changes were more visible in the RT + CS group than in the RT + P group (*p* <  0/05).

**Conclusion:**

Regular resistance training can be recommended as a very suitable non-pharmacological approach to strengthen the body’s antioxidant system, muscle strength and quality of life in older adults. There are no definite findings on the role of creatine on the antioxidant system and quality of life in older adults, but the use of this supplement in addition to RT can double the amount of strength gained from resistance training.

## 1. Introduction

The production of free radicals increases from the fourth decade of life onwards, and the amount of antioxidant enzymes such as superoxide dismutase (SOD) and glutathione peroxidase (GPX) decreases (1). Reactive oxygen species (ROS) lead to oxidative damage in lipid membranes and DNA through programmed cell death (2). Lipid peroxidation produces substances such as malondialdehyde (MDA) (3), and DNA damage is measured through *8-hydroxydeoxyguanosine* (8-OHdG) (4). One of the factors that plays an important role in the loss of an active lifestyle is the progressive reduction of muscle mass or sarcopenia (5). Muscle mass forms more than 50% of the body weight of young people, while this amount reaches approximately 25% in older adults (6).

Cumulative damage to skeletal muscle and nerve cells in sarcopenia may result from oxidative stress. Sarcopenia could be caused by an increase of endogenous ROS formation in skeletal muscle but the source of ROS in sarcopenic muscle is still relatively unknown. However, an age-associated increase of ROS levels in muscle mass, as a consequence of an upregulation of NADPH oxidase 2 enzyme (NOX_2_), has been reported (7). Moreover, a study by Sullivan-Gunn and Lewandowski (8) has highlighted the role of NOX_2_ enzyme in a healthy mouse model of aging, suggesting that elevated levels of H_2_O_2_ from NOX_2_, as well as the lack of antioxidant protection from catalase and glutathione peroxidase (GPX), carry out a key role in the onset of sarcopenia. The lack of SOD_1_ also causes a reduction of skeletal muscle mass, impairment of neurotransmitter release, and neuronal degeneration in mice (9).

Moreover, irrespective of the mechanism, oxidative stress causes the onset of many types of disease such as cardiovascular disease (CVD) and cancer (10, 11) as well as modulation of cancer treatment-related outcomes (12, 13). Excessive ROS levels have been linked to tumor initiation, growth and progression (14). As mentioned before, the production of ROS increases with age. Therefore, the possibility of getting diseases related to ROS increases in older adults. Therapeutic benefits of creatine supplementation (CS) in some diseases associated with oxidative stress have been confirmed (15, 16).

In the body as a whole, creatine is synthesized in the kidney and in the liver (17). Specifically, the kidney accomplishes the first step of the synthesis, forming guanidinoacetic acid from arginine and glycine. Guanidinoacetic acid is then transported to the liver, where it is converted into creatine with the intervention of the methyl donor S-adenosyl-methionine (17). Creatine monohydrate is a common and popular supplement used by many athletes to improve strength, endurance and athletic performance (18). The antioxidant properties of creatine might be due to the presence of arginine, which disintegrates nitric oxide (19). The NO formed undergoes oxidative degradation to the stable inorganic nitrogen oxides, nitrite (NO_2_), and nitrate (NO_3_), which are detectable in plasma and urine (20).

Resistance training (RT) has the beneficial effects of preventing the complications of aging and leads to improved health through a variety of mechanisms (21). RT has been shown to reduce MDA and hydrogen peroxidase, while boosting GPX and SOD activity (22). On the other hand, in aging, we face a phenomenon called anabolic resistance which reduces the response of muscles to RT (23).

Long-term creatine supplementation along with moderate-intensity resistance and endurance training can probably reduce oxidative stress and increase the antioxidant defense system; however, in the short-term, creatine consumption and its effect on oxidative stress due to endurance exercise is not well known, although it seems that the short-term creatine ingestion possibly reduces oxidative stress due to intense resistance exercise. Considering the antioxidant effects of regular physical activity and creatine, it seems that the combined effect of physical activity and creatine consumption can reduce oxidative stress (12).

Stefani et al. (24) noted that creatine consumption combined with resistance exercise could reduce oxidative stress (reduced lipoperoxidation in plasma, heart and liver, and gastrocnemius). Moreover, this supplement had positive effects on the SOD activity in all groups. Creatine consumption possibly have a synergistic effect with resistance training in modulating SOD activity in the heart (24). Additionally, Rahimi stated that consuming 20 g of creatine per day for 7 days reduces MDA and 8-hydroxy-20 -deoxyguanosine (8-OHdG) after resistance training. Additionally, the antioxidant effects of creatine may be related to its compounds (arginine, glycine and methionine) (25).

Most studies on the effect of resistance training (RT) and creatine supplementation (CS) on oxidant/antioxidant equilibrium have focused on young people (25). To date, the effects of regular RT alone and with CS on various oxidative markers and antioxidant defense system in older adults have not been studied.

## 2. Materials and methods

### 2.1. Ethical considerations

Before the start of the study, a meeting session was held to coordinate and explain the objectives of the project and to mention the possible risks and benefits for the participants. At the end of this session, consent forms for participation in the research were signed by the subjects. The research design was approved by the Ethics Committee of the University of Kurdistan (IR.UOK.REC.1399.010), and was also reviewed and approved at the Clinical Trial Registration Center (IRCT20201121049453N1).

### 2.2. Participants and study design

**Figure 1:**
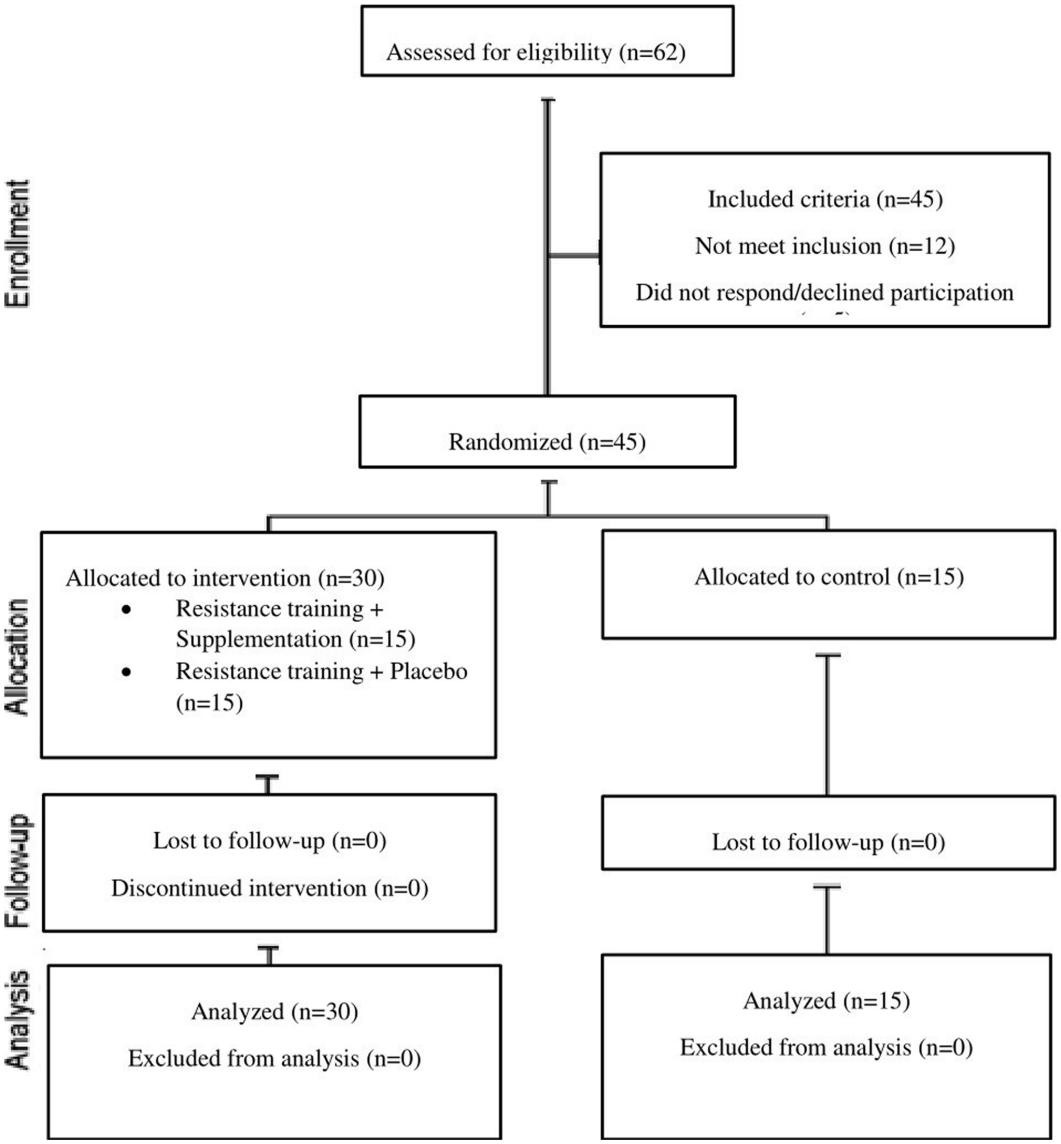
Flowchart of the study.

The current study was an experimental research with pre-test and post-test control group design. The statistical population of the present study consisted of older men and women (age: 61.5 ± 5.7 y; height: 165.9 ± 11.1 cm; weight: 75.6 ± 19.8 kg; BMI: 27.8 ± 1.6 kg/m^2^) in Kermanshah city, Iran. After announcing the research call at parks and sport spaces of the city, 45 qualified people (with inclusion criteria) were randomly selected as subjects. Subjects were then randomly, in a double-blind design, assigned to three groups of 15: resistance training with creatine supplementation (RT + CS), resistance training with placebo (RT + P) and control group (Table 1; Figure 1).

**Table 1:** Baseline characteristics of subjects according to study groups.

Variable	Control group (*n* = 15)	Training + placebo (*n* = 15)	Training + creatine supplement (*n* = 15)
Age (year)	62.5 ± 25.47	61.3 ± 31.57	61.2 ± 0.7.06
Weight (kg)	74.08 ± 18.8	75.37 ± 10.7	77.23 ± 16.9
Height (cm)	168.16 ± 9.50	162.37 ± 6.35	167.38 ± 10.92
BMI (kg/m2)	25.83 ± 5.4	28.37 ± 2.9	27.84 ± 5.1

BMI, body mass index. Values are reported as mean ± SD.

### 2.3. Inclusion and exclusion criteria

People ranging in age from 60 to 70 years were considered. According to the information in the health questionnaire, they were in good health and did not take any medication. They also did not have a history of taking dietary supplements or regular exercise training 3 months prior to the start of the study. Exclusion criteria included any illness that required medical attention, the unwillingness of the subjects to continue cooperation, and having more than three absences in the training sessions (Figure 1).

### 2.4. Resistance training protocol

Resistance training program was performed for 10 weeks, three sessions per week (1 day in between). Each workout lasted approximately 70 mins and consisted of three parts: warm-up (10 mins), specific resistance exercise program (50 mins), and cool-down (10 mins). Resistance movements included leg extension and leg curl machine, barbell bench press, lateral pulldown, barbell curl, overhead press, and triceps extension machine. Resistance movements were performed in three sets with 10 repetitions and an intensity of 75% of one repetition maximum (1-RM). Rest intervals between sets and between movements were 2 and 3 mins, respectively. To maintain training intensity, 1-RM test was measured once every 2 weeks in all movements and for all subjects. The one repetition maximum (1-RM) test was calculated based on the Brzycki equation (26).

### 2.5. Creatine supplementation

Creatine monohydrate supplementation consisted of 0.1 g/kg/d which was consumed daily in a single dose and dissolved in a glass of water immediately after the end of the exercise session by the subjects in the RT + CS group (27, 28). Supplementation on non-training days was consumed at the same hour (6 pm). The placebo group took maltodextrin in the same way as the supplementation group. The control group did not receive any intervention and participated only in pre-test and post-test evaluations.

### 2.6. Sampling and data collection

48 hours before the start of the first training session and creatine supplementation as well as 48 hours after the end of the 10-week course of interventions, blood samples were taken at 8 am in the amount of 6 cc (after approximately 10 hrs of night fasting). The samples were then centrifuged at 3000 g for 10 mins and the extracted serums were frozen at −70°C until final assay.

Malondialdehyde (MDA), glutathione peroxidase (GPX) and total antioxidant capacity (TAC) serum levels were determined using commercial kits (Human ELISA; ZellBio Company, Berlin, Deutschland) according to the manufacturer’s protocol with a lower detection limit of 0.1 μM, 5 U/ml, and 0.1 mM, respectively. The measurement of *8-hydroxydeoxyguanosine* (8OHdG) was performed by a commercial ELISA kit with a lower detection limit of 0.25 ng/ml (BT Lab, Shanghai, China).

As previously mentioned, in order to measure the maximum muscle strength, the Brzycki equation was used (26). Quality of life was measured by the short-form 36 (SF-36) questionnaire (29). This questionnaire comprised of the following sections: physical component score (PCS), mental component score (MCS), physical functioning, role-physical, bodily pain, general health and mental health.

In order to control the participants’ nutritional status, a 24-h dietary recall was taken 48 hrs before the start of both blood sampling steps. In this regard, the calorie intake was analyzed through nutrition software, and there was no significant difference in terms of calorie intake of the subjects in the three groups.

### 2.7. Data analysis

The Shapiro–Wilk test was used to examine the normal distribution of data. Analyses of variance (ANOVA) with repeated measure and Bonferroni post-hoc test were used to determine within-group (time effect), between-group (group effect) and time–group interactions. Data was analyzed with IBM SPSS software (version 23; IBM Corporation, Armonk, NY, United States), and the statistical significance level was set at *p* ≤ 0.05.

## 3. Results

There was no significant difference (*p* > 0.05) between the three groups regarding any of the research variables in the pre-test.

**Figure 2:**
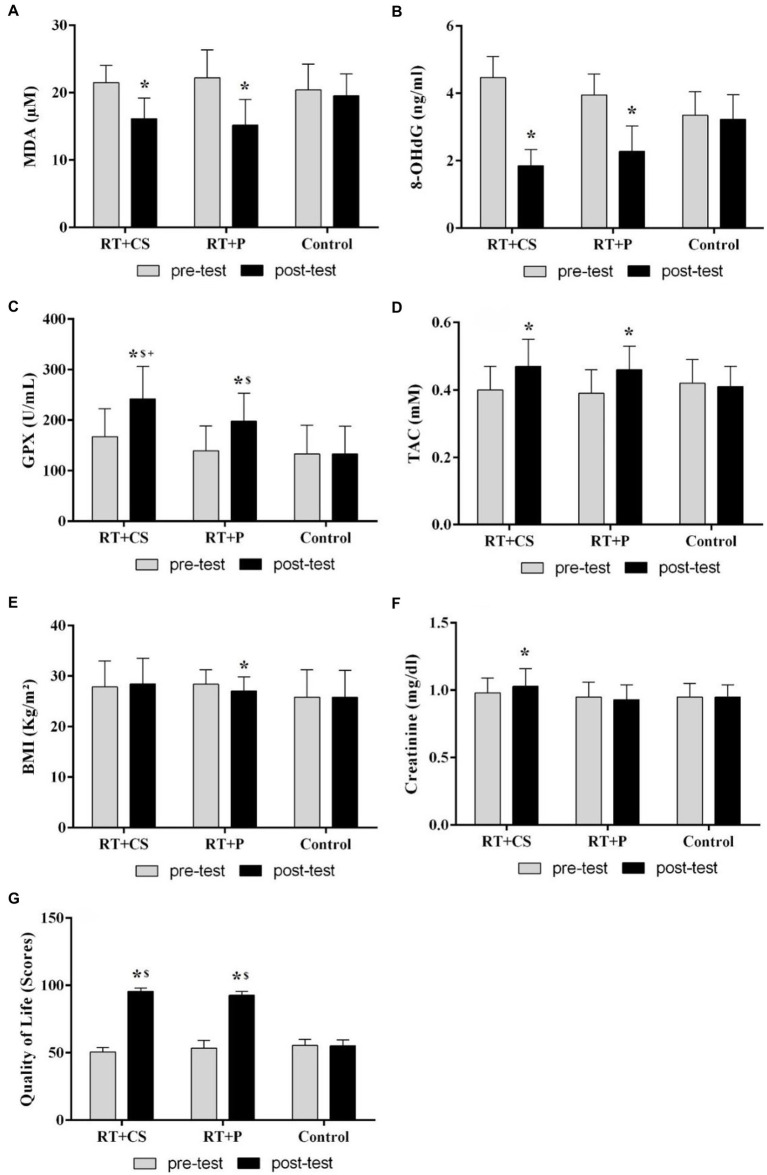
Changes in oxidative and antioxidant indices, as well as creatinine, BMI and quality of life in the older adults after 10-week of resistance training and creatine supplementation. **(A)** Malondialdehyde (MDA); **(B)** *8-hydroxydeoxyguanosine* (8-OHdG); **(C)** Glutathione peroxidase (GPX); **(D)** Total antioxidant capacity (TAC); **(E)** Body mass index (BMI); **(F)** Creatinine; **(G)** Quality of life. *Significant difference with the pre-test. ^$^Significant difference with the control group.

There were significant main effect of time (*F* = 127.38, *p* = 0.001, and *F* = 468.47, *p* = 0.001) and group × time interaction (*F* = 21.98, *p* = 0.001, and *F* = 106.21, p = 0.001) respectively for malondialdehyde (MDA) and *8-hydroxydeoxyguanosine* (8-OHdG). These serum indices of oxidative damage were significantly reduced in both experimental groups (RT + CS and RT + P) after 10 weeks of RT (in all cases, *p* = 0.001) and there was no difference between the training groups (*p* > 0.05; Figures 2A,B).

In relation to the glutathione peroxidase (GPX), there was a significant main effect of time (*F* = 155.32, p = 0.001), group × time interaction (*F* = 37.47, *p* = 0.001) and main effect of group (*F* = 5.39, *p* = 0.009). Serum concentrations of GPX was higher in both training groups (RT + CS and RT + P) compared with the control group in the post-test (*p* = 0.001 and *p* = 0.006, respectively). Indeed, in the post-test, the levels of this enzyme in the RT + CS was higher than the RT + P group (*p* = 0.004). GPX substantially increased in the RT + CS and RT + P groups after the 10-week intervention compared with the pre-test (*p* = 0.001, eta = 0.87 and *p* = 0.001, eta = 0.86; Figure 2C).

In regards total antioxidant capacity (TAC), there was a significant main effect of time (*F* = 41.77, *p* = 0.001) and group × time interaction (*F* = 15.51, *p* = 0.001). This index of antioxidant capacity also had an interesting rise in both training groups after 10 weeks (in both cases, *p* = 0.001), while no significant difference was observed between the groups in the post-test (*p* > 0.05; Figure 2D).

Regarding body mass index (BMI), there was a significant main effect of time (*F* = 3.68, *p* = 0.049) and group × time interaction (*F* = 23.93, *p* = 0.001). Intragroup changes showed that the body mass index decreased (*p* = 0.001) only in the RT + P group in comparison with the pre-test (Figure 2E).

However, with regard to creatinine, the interaction of time and group was significant (*F* = 6.40, *p* = 0.004). Creatinine levels elevated only in the RT + CS group after 10 weeks of supplementation (*p* = 0.014). No significant difference was observed between the groups in the post-test (*p* > 0.05; Figure 2F).

There was also a significant main effect of time (*F* = 1831.58, *p* = 0.001), group × time interaction (*F* = 441.69, p = 0.001) and main effect of group (*F* = 111.97, *p* = 0.001) for quality of life (Figure 2G). This index in the experimental groups (RT + CS and RT + P) improved after 10 weeks of intervention (in both groups, *p* = 0.001). Indeed, there was a difference between the groups in the post-test so much so that the quality-of-life index in the training groups was better than in the control group (Table 2).

**Table 2:** Changes in subjects’ quality of life and muscle strength following 10-week of resistance training and creatine supplementation.

		Groups	Pre-test (M ± SD)	Post-test (M ± SD)	Repeated Measure	*f*	*p*
Muscle strength (kg)	Leg extension	RT + CS RT + P Control	33.61 ± 36.38	50.53 ± 52.61	Time Group Time × Group	20.57	0.001*
			16.87 ± 23.50	22.87 ± 23.58		2.01	0.14
			26.91 ± 5.53	26.83 ± 5.16		8.25	0.001*
	Leg curl	RT + CS RT + P Control	15.92 ± 14.66	29.46 ± 26.73	Time Group Time × Group	26.38	0.001*
			9.62 ± 9.16	14.43 ± 12.69		3.59	0. 03*
			9.08 ± 3.34	9.08 ± 3.22		10.40	0.001*
	Barbell bench press	RT + CS RT + P Control	32.15 ± 21.83	47 ± 31.53	Time Group Time × Group	35.81	0.001*
			19.62 ± 9.81	25.68 ± 14.40		4.14	0.024*
			23.83 ± 6.75	23.58 ± 6 ± 43		13.50	0.001*
	Lat pulldown	RT + CS RT + P Control	38.76 ± 10.96	57 ± 16.03	Time Group Time × Group	77.50	0.001*
			31.18 ± 6.24	40.87 ± 6 ± 81		19	0.001*
			27.58 ± 4.16	27.16 ± 3.80		24.87	0.001*
	Barbell curl	RT + CS RT + P Control	19.38 ± 10 ± 73	30.07 ± 15.56	Time Group Time × Group	46.70	0.001*
			14.87 ± 7.67	18.56 ± 10.54		6.01	0.005*
			11.75 ± 3.98	11.58 ± 4.01		19.75	0.001*
	Overhead press	RT + CS RT + P Control	8.84 ± 4.01	15.46 ± 6.67	Time Group Time × Group	80.19	0.001*
			6.93 ± 3.19	10.43 ± 4.42		6.31	0.001*
			6.83 ± 1.74	6.66 ± 1.82		25.89	0.004*
	Triceps extension	RT + CS RT + P Control	32.61 ± 15.56	50.30 ± 22.62	Time Group Time × Group	76.66	0.001*
			24.12 ± 6.35	32.06 ± 10.89		8.24	0.001*
			22.25 ± 5.59	22.25 ± 5.59		35.59	0.001*
Quality of life (scores)		RT + CS RT + P Control	50.50 ± 3.58	95.61 ± 2.34	Time Group Time × Group	1831.58	0.001*
			53.59 ± 5.59	92.70 ± 2.79		111.97	0.001*
			55.45 ± 4.59	55.20 ± 4.25		441.69	0.001*

RT + CS resistance training with creatine supplementation. RT + P resistance training with placebo.

* Significant difference at *p* < 0.05.

Changes in muscle strength in all seven resistance movements including leg extension and leg curl machine, barbell bench press, lateral pulldown, barbell curl, overhead press, and triceps extension machine indicated that the training groups (RT + CS and RT + P) had a sharp increase compared to the pre-test (*p* < 0.05) as well as compared to the control groups in the post-test (*p* < 0.05). In addition, the increase in muscle strength of the RT + CS group was greater than that of the RT + P group (*p* < 0.05; Table 2).

## 4. Discussion

Findings of the present study showed that 10 weeks of RT reduced the oxidative damage indices in older adults by strengthening the antioxidant defense system. In fact, after 10 weeks of regular RT, MDA and *8-hydroxydeoxyguanosine* (8-OHdG) in the training groups decreased, while the amount of GPX and total antioxidant capacity (TAC) increased. An interesting result is that creatine supplementation did not have an incremental effect on reducing oxidative damage. However, the rate of increase in GPX enzyme in the supplement group was higher than the placebo group demonstrating the synergistic effect of creatine supplementation on the levels of this antioxidant enzyme.

Alikhani and colleagues in line with the present study illustrated an improvement in MDA and TAC in older and younger people after 12 weeks of RT. (30) Consistent with the current findings, two other studies also confirmed the positive effects of regular exercise training on improving the activity of antioxidant enzymes such as catalase, superoxide dismutase and glutathione peroxidase in the older adults (31, 32). The effect of combined training (aerobic and resistance training) on DNA oxidative damage and antioxidant properties in middle-aged and older people was investigated by (33) which indicated the positive role of the above exercise program in improving antioxidant capacity and reducing 8-OHdG. Ghahramani Moghadam^’^s study also demonstrated a reduction in 8-OHdG levels in older women after 8 weeks of exercise training (34). In another study by Padilha, three sessions of RT per week (for 12-weeks) reduced oxidative stress indices in older women (35). In a research conducted by Koechlin, the effect of RT with an intensity of 40% of 1-RM with N-acetylcystein supplementation on the older adults with chronic obstructive pulmonary disease was evaluated and indicated that the TAC index did not change (36). It appears that the low intensity of the exercise program, the type of supplement used and also the illnesses of the subjects are the reasons for the contradiction in the findings of the above research with the results of the present study.

Kinksly et al. examined the effect of cycling and creatine consumption on non-enzymatic indicators of antioxidant defense. The finding of the above study indicated that creatine had no effect on improving the antioxidant system (37). However, in a study conducted on young subjects stated that 7 days of creatine supplementation reduced MDA and 8-OHdG following acute RT. Candow et al. reported that creatine supplementation improved body composition and muscle strength in older adults without any adverse effects on the kidney (27). Canadow in another study illustrated that creatine supplementation had anti-sarcopenic effects and would improve bone mineral density (38). In addition, Deminice et al. stated that short-term creatine supplementation (for 7 days) had no effect on catalase and superoxide dismutase activity after an anaerobic test (39).

According to the results of previous research, it seems that short-term use of creatine supplementation has no effect on strengthening the antioxidant system. Available findings are limited and relatively contradictory in regards the long-term effects of this supplement which makes the final conclusion difficult. As mentioned, in the present study, 10 weeks of creatine monohydrate supplementation doubled the activity of GPX enzyme, while no synergistic effect of this supplement on TAC were observed. Therefore, further research is needed to prove the possible effects of creatine on boosting the antioxidant system. Nevertheless, it has been found that regular RT can be a logical approach for reducing exercise training-induced oxidative damage by enhancing the antioxidant capacity of older adults.

The finding of the present study regarding changes in muscle strength showed that after 10 weeks of RT and creatine supplementation, the average increase in muscle strength (mean of the seven muscle groups studied) in the training-placebo group was 35.9% and in the training-creatine group was equal to 57%. These findings generally indicate the positive effect of RT on increasing muscle strength in the older adults, and in particular the synergistic effects of creatine supplementation on elevating strength in these individuals. Moreover, the results of the current study demonstrated that there was a significant decrease in body mass index only in the training-placebo group. The present research finding have been confirmed by previous research on the effect of RT on upgrading muscle strength and body composition, as well as the dual role of creatine supplementation in improving muscle strength in older adults (27).

The present results showed an improvement of 79.9% and 89.2% of quality of life index in the training-placebo and training-creatine groups, respectively.

### 4.1. Limitations

To our knowledge, the present study is the first study that examines the long-term effect of resistance training along with creatine supplementation on antioxidant indices in older adults. One of the limitation of the current study include the lake of measurement of nitric oxide metabolites. In addition, it is better to measure the level of muscle tissue at the same time as blood sampling in future studies to check the changes in oxidant/antioxidant indices more precisely.

## 5. Conclusion

Regular resistance training can be recommended as a very suitable non-pharmacological approach to strengthen the body’s antioxidant system, muscle strength and quality of life in older adults. There are no definite findings on the role of creatine on the antioxidant system and quality of life in older adults, but the use of this supplement in addition to RT can double the amount of strength gained from resistance training. From a clinical viewpoint, our findings indicated that these results can be obtained using a modest exercise prescription for an aging population.

## Data availability statement

The raw data supporting the conclusions of this article will be made available by the authors, without undue reservation.

## Ethics statement

The studies involving human participants were reviewed and approved by the research design was approved by the Ethics Committee of the University of Kurdistan (IR.UOK.REC.1399.010). The patients/participants provided their written informed consent to participate in this study.

## Author contributions

EA: writing original draft of the manuscript. DS-V: methodology, editing the manuscript, and supervision. EA and DS-V: data analysis and interpretation. All authors contributed to the article and approved the submitted version.

## Conflict of interest

The authors declare that the research was conducted in the absence of any commercial or financial relationships that could be construed as a potential conflict of interest.

## Publisher’s note

All claims expressed in this article are solely those of the authors and do not necessarily represent those of their affiliated organizations, or those of the publisher, the editors and the reviewers. Any product that may be evaluated in this article, or claim that may be made by its manufacturer, is not guaranteed or endorsed by the publisher.
